# Contribution of cropland to the spread of Shiga toxin phages and the emergence of new Shiga toxin-producing strains

**DOI:** 10.1038/s41598-017-08169-6

**Published:** 2017-08-10

**Authors:** Pablo Quirós, Maite Muniesa

**Affiliations:** Department of Genetics, Microbiology and Statistics, University of Barcelona, Diagonal 643, Annex, Floor 0, E-08028 Barcelona, Spain

## Abstract

A growing interest in healthy eating has lead to an increase in the consumption of vegetables, associated with a rising number of bacterial outbreaks related to fresh produce. This is the case of the outbreak in Germany, caused by a O104:H4 enteroaggregative *E*. *coli* strain lysogenic for a Stx phage. Temperate Stx phages released from their hosts occur as free particles in various environments. This study reports the occurrence of Stx phages in vegetables (lettuce, cucumber, and spinach) and cropland soil samples. Infectious Stx2 phages were found in all samples and many carried also Stx1 phages. Their persistence in vegetables, including germinated sprouts, of Stx phage 933 W and an *E*. *coli* C600 (933 W∆*stx*::*gfp*-*cat*) lysogen used as surrogate, showed reductions below 2 log_10_ units of both microorganisms at 23 °C and 4 °C over 10 days. Higher reductions (up to 3.9 log_10_) units were observed in cropland soils at both temperatures. Transduction of a recombinant 933 W∆*stx*::*kan* phage was observed in all matrices. Protecting against microbial contamination of vegetables is imperative to ensure a safe food chain. Since the emergence of new Stx strains by Stx phage transduction is possible in vegetable matrices, methods aimed at reducing microbial risks in vegetables should not neglect phages.

## Introduction

Shiga toxin-producing *Escherichia coli* (STEC) are foodborne pathogens associated world wide^1^ with human diseases such as diarrhea, hemorrhagic colitis, hemolytic uremic syndrome (HUS), and thrombotic thrombocytopenic purpura (TTP)^2^. The principal virulence factor of STEC is the Shiga toxin. There are two Stx types, Stx1 with 4 subtypes (a, c, d, and e) and Stx2 with 7 subtypes (a to g)^3, 4^. In humans, Stx2 is the most dangerous type and is often associated with HUS^5^. In mouse assays, Stx2 was 400 times more virulent than Stx1^6^.

The genes encoding Stx in *E*. *coli* are located in the genomes of inducible temperate bacteriophages (Stx phages)^7^. *stx* genes are overexpressed when the lytic cycle of the phage is induced^8^. A bacterial SOS system is activated when bacteria detect environmental stressors such as antibiotics, chemicals, ultraviolet light, or salts, triggering the switch from a lysogenic to a lytic cycle^9, 10^. In addition, the bacterial lysis caused by Stx phages leads to the dissemination, propagation, and excretion of these phages through feces^11^.

Domestic ruminants comprise the main reservoir of STEC^12, 13^. The ingestion of contaminated food or water and direct contact with rural environments are the most common routes of transmission to humans. Recent years have witnessed a global trend toward eating a healthy diet rich in fruits, vegetables, and fresh, raw vegetable products. However, these vegetables may have been grown in soil in open fields, entailing a risk of contamination by toxins or pathogens^14^. Moreover, soil, manure, and irrigation water are important sources of plant contamination^15, 16^. At all these stages, the interactions between vegetables and enteric pathogens may be more complex than previously thought.

There have been numerous Stx-producing *E*. *coli* outbreaks linked to vegetable consumption. One of the most notable was the widespread outbreak in Japan associated with sprouts in 1996^17^. A growing number of foodborne illnesses have been increasingly traced back to fruits and vegetables in the last ten years^18–21^, arousing concern that plants might be an important vehicle for human enteric pathogens.

In 2011, there was a major outbreak in the European Union (Germany and France) of STEC O104:H4 traced to sprouted fenugreek seeds produced in Egypt, in which 3,816 people were infected, 845 developed HUS and 54 died^22, 23^. The causative agent of this outbreak was an enteroaggregative *E*. *coli* strain of serotype O104:H4 with the capacity to produce Shiga toxin through the transduction of *stx*_2a_ via a temperate Stx phage^24, 25^. The origin of this Stx phage and the circumstances leading to the transduction event are unknown.

Free Stx phages are more persistent than bacteria^26^ and are involved in the horizontal transduction of *stx* ^27, 28^. Their persistence and likelihood of transduction increase the risk of new STEC strains emerging in the environment and in food, particularly when food is consumed raw or only partially cooked. The aim of this study was to evaluate the occurrence, persistence, and capacity of Stx phages to transduce *stx* and generate Stx-lysogens using different types of salad vegetable and cropland soil as matrices.

## Results

### Detection of Stx1 and Stx2 phages in vegetable and soil matrices

Stx phages were quantified by qPCR in lettuce, cucumber, spinach, and soil samples and positive samples were considered when the qPCR threshold cycle (Ct) was below 32 (quantification limit of 38.7 GC/well). Some samples showed negative amplification (undetermined), while some showed amplification signal but with Ct values above 32, and were considered negatives. Stx1 phages were rarely present in the samples analyzed, and were only detected in one cucumber sample. In contrast, Stx2 phages were present in soil, lettuce, and cucumber (Table 1), and only in spinach did values fall below the quantification limit. When detected, the densities (GC.g^−1^) of the Stx phages were not negligible, being in average above 10^3^ GC.g^−1^ in all positive samples (Table 1).

**Table 1:** Occurrence of Stx1 and Stx2 phages (GC.g^−1^) in vegetable and soil samples.

n	Soil	Lettuce	Cucumber	Spinach
	16	15	15	15
Stx1 phages*	0	0	5.6 × 10^4^	0
n° of positives samples	0	0	1	0
Stx2 phages*	9.5 × 10^3^ (9.7 × 10^3^)	6.8 × 10^3^	3.8 × 10^4^ (1.1 × 10^4^)	0
n° of positives samples	8	1	2	0

*Average value of the positive samples. In brackets SD.

### Propagation of Stx phages in the samples

**Figure 1:**
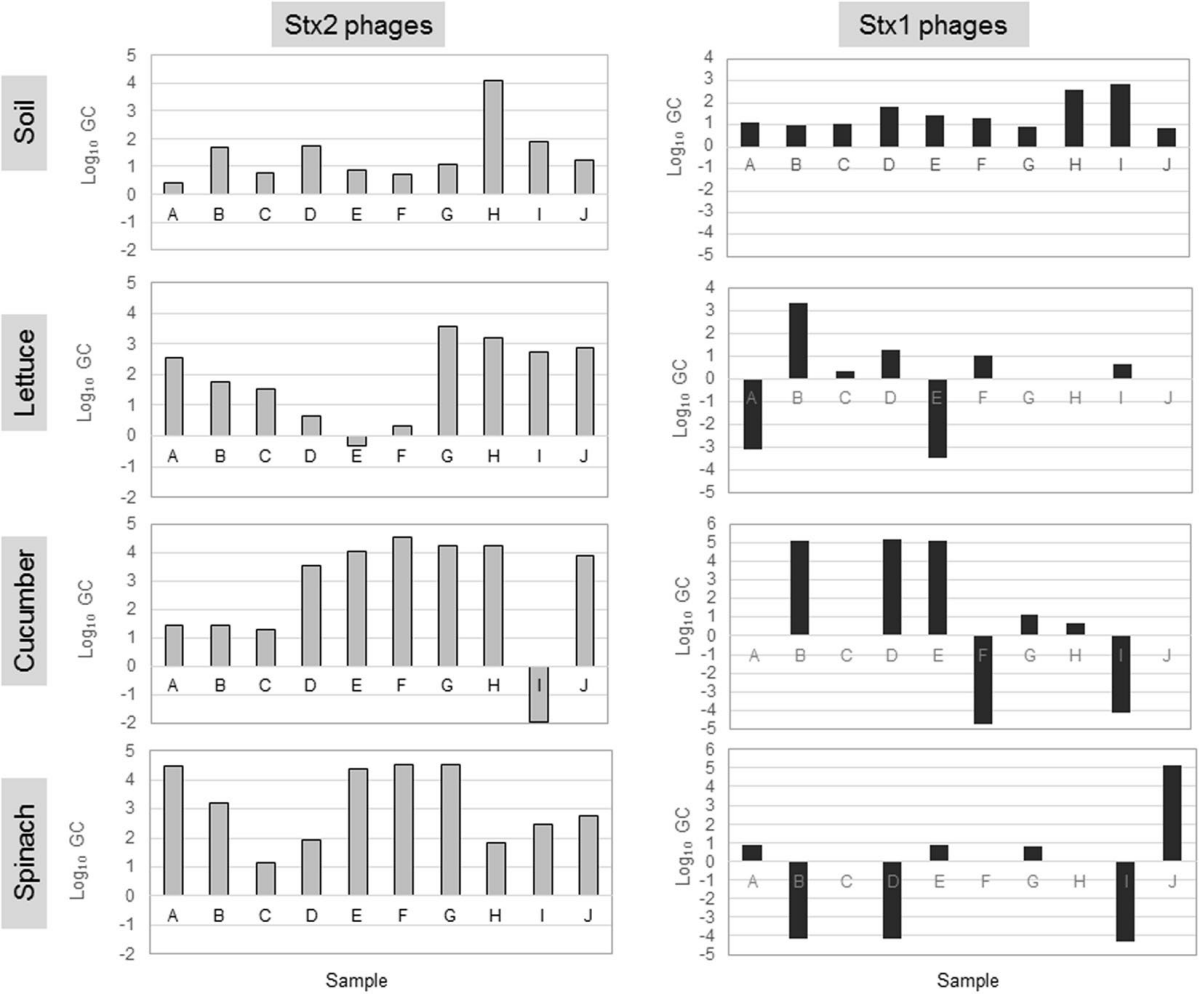
Difference in log_10_ GC units of Stx1 and Stx2 phages extracted from matrices after propagation in *E*. *coli* WG5 strain. A positive difference indicates phages were infective and able to propagate.

To evaluate the infectivity of Stx phages naturally occurring in the samples, phage particles purified from ten samples (from A to J) of each matrix, each from a different location, were used to infect a culture containing *E*. *coli* WG5 strain, previously reported as being sensitive to a wide range of Stx1 and Stx2 phages^29^. This approach offers two advantages for Stx phage detection: it generates information about the infectivity of the Stx phage particles, because of their capacity to propagate and, as shown in Fig. 1, it enables detection of Stx phages after propagation in samples that were otherwise negative if directly analyzed. We observed a significant (P < 0.05) increase (in log_10_ GC units) in the densities of Stx phages after their propagation in many of the samples (Fig. 1), particularly for Stx2 phages and in a lesser extent for Stx1 phages. The increase ranged from less than 1 log_10_ units to up to 4–5 log_10_ GC (Fig. 1).

### Persistence of Stx2 phages in the samples

Phage 933W, considered a model for the study of Stx phages, was used to evaluate persistence in the vegetable and soil samples. Simultaneously, and to avoid bias caused by different samples, we evaluated the persistence of *E*. *coli* C600 strain lysogenic for a recombinant phage 933W, in which the *stx* genes were replaced by *gfp-cat* genes (Fig. S1). This modification made it possible to quantify bacterial cells (by *gfp* absolute qPCR quantification) and Stx phages (by *stx*_2_ absolute qPCR quantification) in the same samples and under the same conditions, using the two qPCR assays (Table 2). Viable bacterial cells grown in LB agar plates + cm and infectious Stx phages with the capacity to infect *E*. *coli* WG5 enumerated by plaque blot were also monitored.

**Table 2:** Oligonucleotides used in this study.

Name	Sequence (5′- 3′)	Target	Reference
S2Aup	ATGAAGTGTATATTATTTA	*stx*_2A_ fragment	66
S2Alp	TTCTTCATGCTTAACTCCT		
gfp short-up	TCCATCTTCAATGTTGTGTCT	*gfp* fragment	67
gfp short-lp	GAACTATAAGACACGTGCTGA	*gfp* fragment	
km-5	GTCAGCGTAATGCTCTGC	fragment of *kan* in pOX38	This study
km-3	GTCTGCTTACATAAACAG		This study
km5-stx	GCGTTTTGACCATCTTCGTCTGATTATTGAGCAAAATAATTTATATGTGGCCGGGTCAGCGTAATGCTCTGC	Amplifying *kan*. Underlined homology with *stx*2A overlapping *stx*2A	This study
km3-stx	ACAGGAGCAGTTTCAGACAGTGCCTGACGAAATTCTCTCTGTATCTGCCTGAAGTCTGCTTACATAAACAG	Amplifying *kan*. Underlined homology with *stx2A*	This study
rho	TCATCGGGACAGAGCGCCA	upstream stx2-A	68, 69
RR46 LP	GAGCTCTAAGGAGGTTAT	red recombinase in pKD46	61
RR46-UP	GTGCAGTACTCATTCGTT		
GFPTir-F	GCTTCCATCTTCAATGTTGTGTCT	real-Time qPCR for *gfp*	67
GFPTir-R	CATTCTTGGACACAAATTGGAATACAACT		
GFPTir-probe	FAM-CATGGCAGACAAACAA-NFQ		
stx1up qPCR	GCGGTTACATTGTCTGGTGACA	real-Time qPCR for *stx*_1_	49
stx1lp qPCR	GCATCCCCGTACGACTGATC		69
stx1-probe	FAM-TAGCTATACCACGTTACAGCG-NFQ		
STX-Any f	ACGGACAGCAGTTATACCACTCT	real-Time qPCR for *stx*_2_	
STX-Any r	CTGATTTGCATTCCGGAACGT		
STX-Any probe	FAM-CCAGCGCTGCGACACG-NFQ		

**Figure 2:**
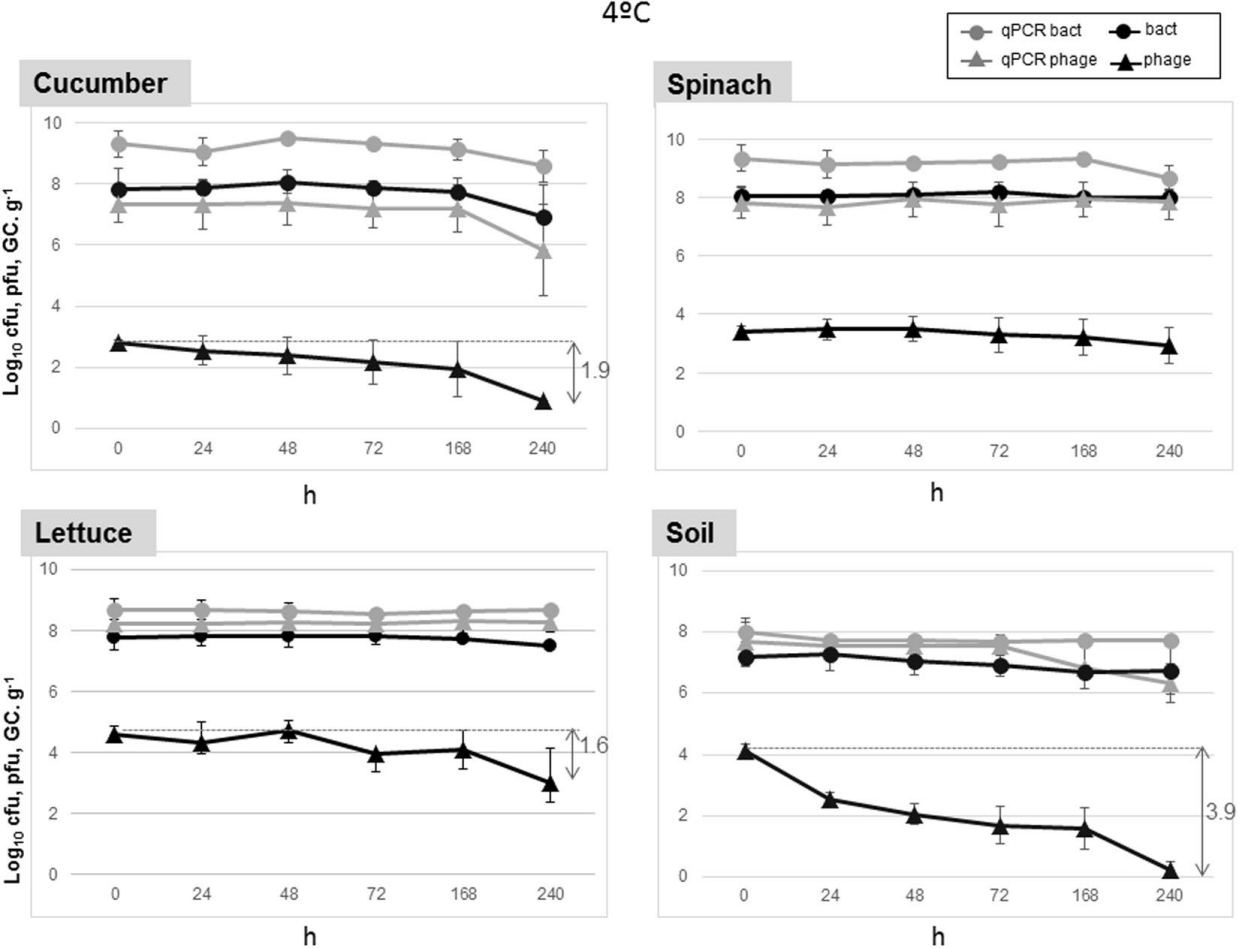
Stability of 933W and *E*. *coli* C600 (933W∆*stx*::*gfp*-*cat*) after 10 days of storage at 4 °C in the different matrices (cucumber, spinach, lettuce, and soil). Values for culturable bacteria are expressed in log_10_ cfu. g^−1^. Infectious phages were evaluated by plaque blot and values expressed as log_10_ pfu g^−1^. Phage and bacterial genomes were evaluated by qPCR with the *stx* qPCR assay and the *gfp* qPCR assay, respectively, and the results are expressed as log_10_ GC.g^−1^. All results are the mean of three independent experiments. Those microorganisms showing significant (*P* < 0.05) reductions are indicated by a vertical arrow.

**Figure 3:**
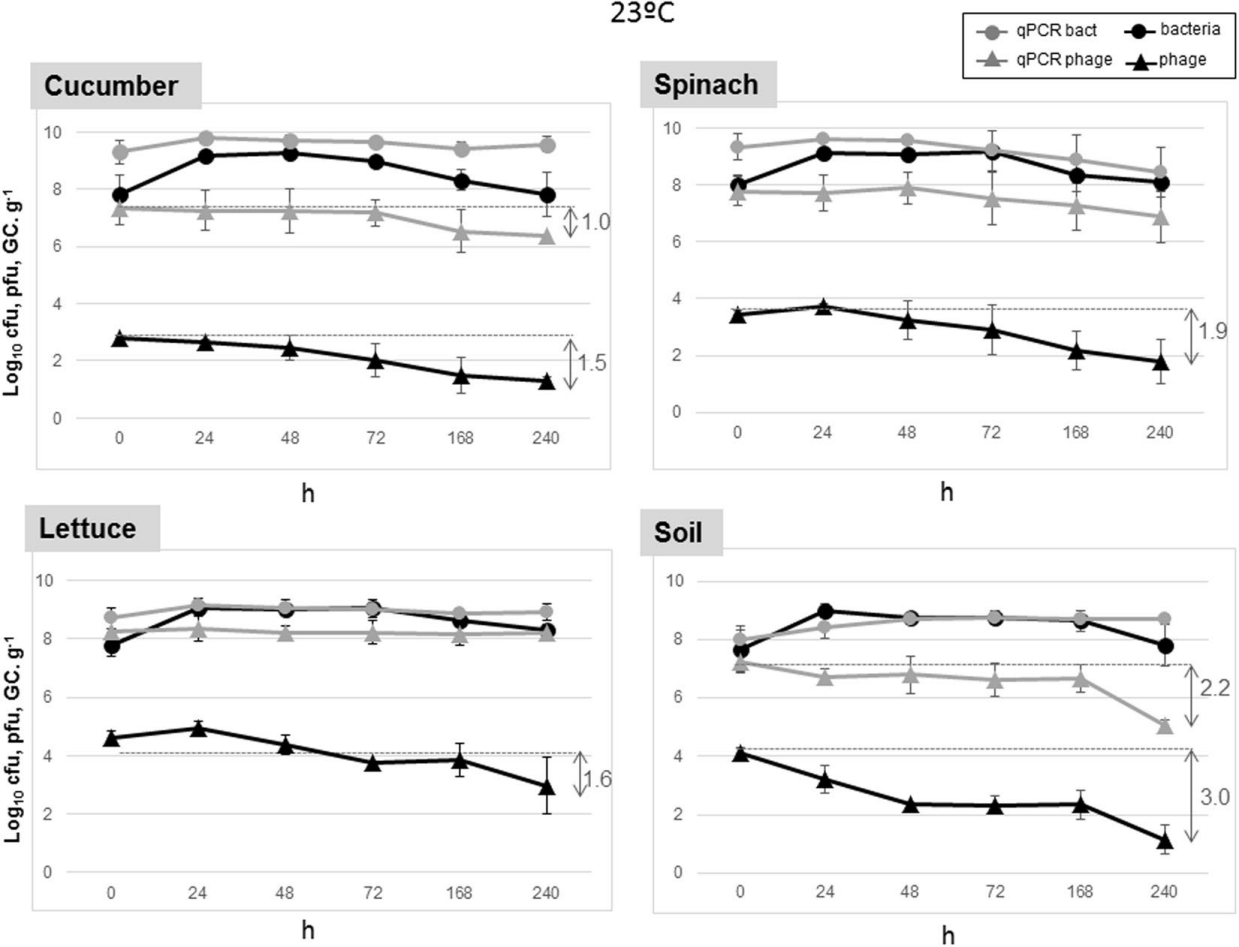
Stability of 933W and *E*. *coli* C600 (933W∆*stx*::*gfp*-*cat*) after 10 days of storage at 23 °C in the different matrices (cucumber, spinach, lettuce, and soil). Values for culturable bacteria are expressed in log_10_ cfu.g^−1^. Infectious phages were evaluated by plaque blot and values expressed as log_10_ pfu g^−1^. Phage and bacterial genomes were evaluated by qPCR with the *stx* qPCR assay and the *gfp* qPCR assay, respectively, and the results are expressed as log_10_ GC.g^−1^. All results are the mean of three independent experiments. Those microorganisms showing significant (*P* < 0.05) reductions are indicated by a vertical arrow.

Similar trends in persistence in soil, cucumber, lettuce, or spinach were observed at 4 °C (Fig. 2) and 23 °C (Fig. 3), both assayed as being common storage temperatures for vegetables.

When bacteria were analyzed by qPCR, reductions in the number of GC were not significant (*P* > 0.05) and lower than 1 log_10_ unit under all conditions. In contrast, the qPCR assay for phages in cucumber and soil at 23 °C showed moderate but significant (*P* < 0.05) reductions of more than 1 log_10_ unit, in accordance with the reductions shown by infectious phages (Figs 2 and 3) over the 10 days that the experiments lasted. The results were repeated in the three replicates performed independently.

The reduction in culturable bacteria under all conditions and in all matrices was very low (less than 1 log_10_ unit) and no significant (*P* > 0.05). In this situation, bacterial counts could also be influenced by cell regrowth during storage. This was particularly marked at 23 °C, a temperature at which the curves showed a significant (*P* < 0.05) increase (black dots), initiated in the first 24 hours and probably maintained during the 10 day period, compensating for cell death (Fig. 3). This growth was not observed at 4 °C (Fig. 2) but also there was not a significant (*P* < 0.05) reduction in viable bacteria. In contrast, the number of infectious Stx phages (and to a lesser extent, their counterpart qPCR values as indicated above) significantly (*P* < 0.05) dropped off during storage at either 4 °C or 23 °C (Figs 2 and 3). A comparison between the number of infectious Stx phages and qPCR values revealed large differences with the molecular quantification. The reduction in infectious Stx phages was most marked in soil samples and the lowest reductions were observed in spinach at 4 °C (only 0.5 logs).

**Figure 4:**
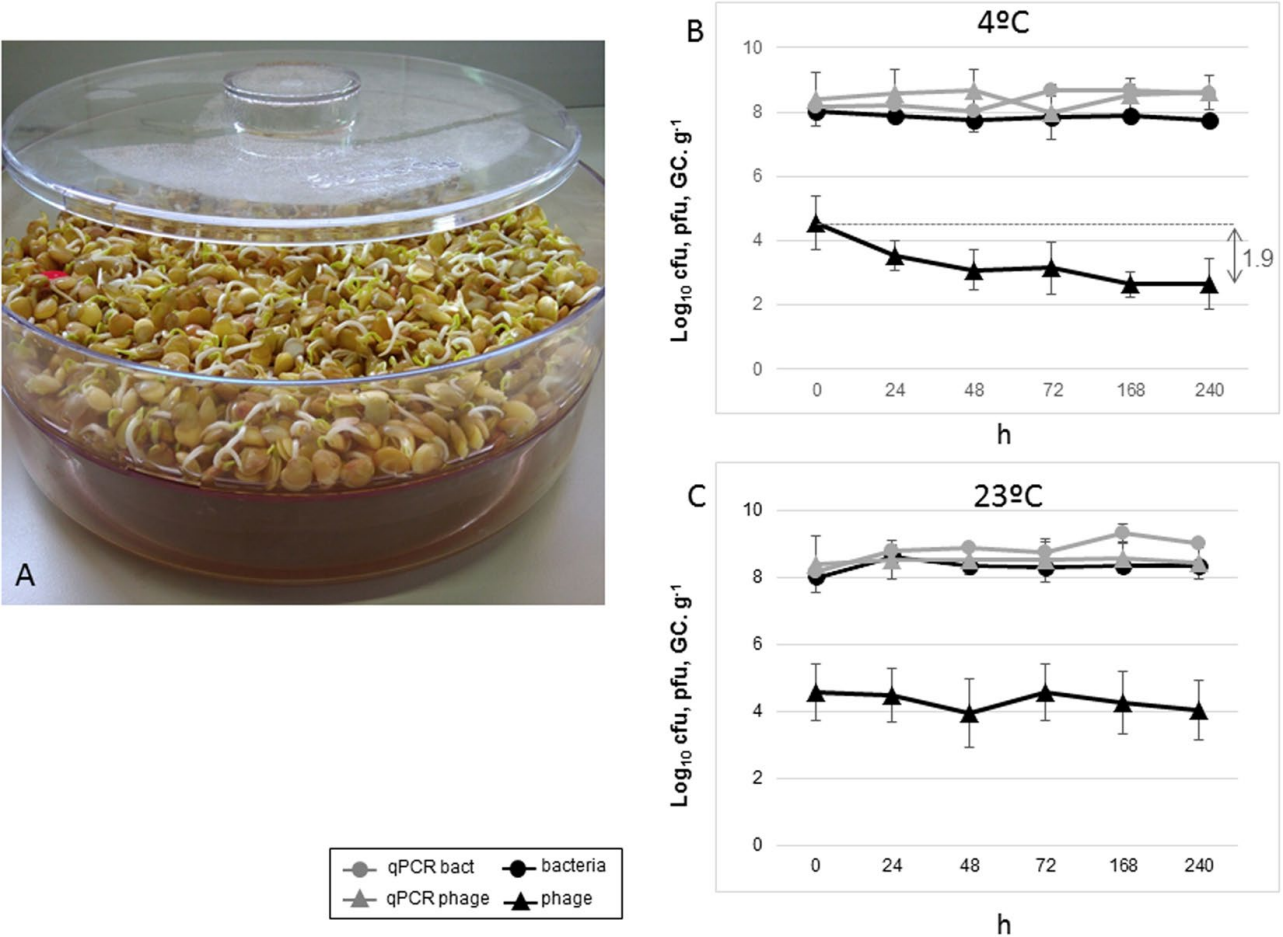
(**A**) Laboratory production of lentil sprouts in the germinator at 23 °C. (**B**) Stability of 933W and *E*. *coli* C600 (933W∆*stx*::*gfp*-*cat*) in the sprouts after 10 days of storage at 4 °C. (**C**) Stability of 933W and *E*. *coli* C600 (933W∆*stx*::*gfp*-*cat*) in the sprouts after 10 days of storage at 23 °C. Culturable bacteria (log_10_ cfug^−1^). Infectious Stx phages (log_10_ pfug^−1^). Phage and bacterial genomes were evaluated by qPCR (log_10_ GC.g^−1^). All results are the mean of three independent experiments. For each replicate, different sprouts were produced and used. Those microorganisms showing significant (*P* < 0.05) reductions are indicated by a vertical arrow.

We sprouted lentil seeds in laboratory conditions using sterile water to perform a similar series of persistence experiments at both temperatures and for both microorganisms (Fig. 4). When cultivated in sprouts, only a significant (*P* < 0.05) reduction of infectious Stx phages was observed at 23 °C. However, this result was not in correspondence with qPCR values, that did not decrease. This lack of correspondence between infectious Stx phages and qPCR evaluation was also observed in lettuce, but not in the other matrices. Bacteria (culturable and qPCR) showed a moderate (0.4 and 0.7 log units) but still significant (*P* < 0.05) increase at 23 °C, attributed as explained above, to the growth of the strain (Fig. 4).

Long-term storage of Stx phages was additionally assayed with ten soil samples stored at 4 °C for six months. After storage, the ten samples were negative for Stx1 phages and 6/10 were positive for Stx2 phages (average values of 1.5 × 10^4^ GC.g^−1^ (SD of 8.6 × 10^3^)). Phages were propagated in *E*. *coli* WG5 and after propagation 3/10 samples showed Stx1 phages (average values of 2.6 × 10^5^ GC.g^−1^ (SD of 2.1 × 10^5^)). 10/10 samples showed Stx2 phages (average values of 1.3 × 10^5^ GC.g^−1^ (SD of 1.2 × 10)). However, evaluation of infectious phage particles in these samples was not possible because no plaques of lysis were detected by plaque blot hybridization.

### Transduction of Stx phages in vegetable and soil matrices

Infectious Stx phages have the potential capacity to infect a suitable bacterial host and convert it into a Stx producer. The *S*. *sonnei* strain 866 was used to evaluate the transduction of Stx phages within the matrices. This host has previously been reported to be a very efficient host for Stx phage transduction, and also shows natural resistance to tetracyclin (Tc). Transduction was successfully achieved using a recombinant phage 933W in which *stx* was replaced by the kanamycin (Km) resistance gene (933WΔ*stx*_2_::*kan*) (Fig. S1), enabling selection of transductants and reducing the interference of background flora present in the samples by inhibiting its growth in the LB agar plates supplemented with Km and Tc (see materials and methods).

**Figure 5:**
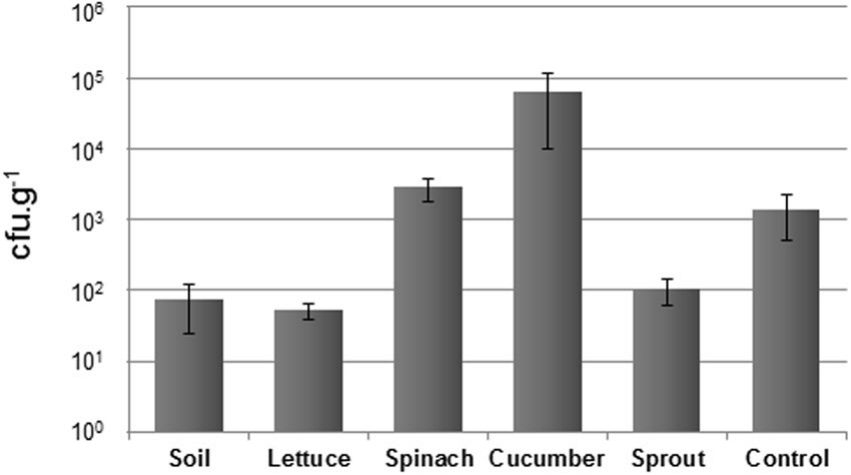
Transductants (cfu.g^−1^) of phage 933W∆*stx*::*km* in *S*. *sonnei* 866 generated in soil, lettuce, spinach, cucumber, and sprouts, and in LB broth at 23 °C. Results are the mean of four independent experiments.

Transductant colonies were generated in all matrices assayed, with a significant (P < 0.05) higher number of transductants observed in cucumber (Fig. 5), followed by spinach, and in lower amounts in soil, lettuce, and lentil sprouts. The control performed in LB showed no significant (P > 0.05) differences with the spinach matrices and deviations comparable to those observed in the samples. These deviations between replicates were attributable to the different efficiencies in generation and growth of the transductants, in variations in the rate of induction of the phage in each replicate or the different moment during the incubation of the culture in which transductants were generated. Nevertheless, the number of transductants counted in the agar plates was the result of 12 h incubation in the matrices at 23 °C. The number of transductants obtained allow to observe differences between matrices, but because they were produced after an incubation period, the qualitative rather than the quantitative result should be considered.

At least 20% of the km-resistant transductants generated with the colony morphology of *Shigella* were confirmed by PCR amplification using rho-km3 as primers (Table 2). All colonies tested positive and were confirmed as transductants, indicating that the interference of background flora in the matrices was negligible. Controls performed with *S*. *sonnei* strain 866 without the 933W-km phage did not show colonies in the presence of Km-Tc, confirming the lack of false positive colonies.

## Discussion

Consumption of fresh produce is rising, mainly due to heightened awareness of the benefits of eating such products and the health education campaigns that several governments have carried out promoting the consumption of fruit and vegetables^30^. This has led to consumer demand for increased choice, including minimally processed, pre-packed, ready-to-eat fruit and vegetables, out-of-season produce, and exotic products^31^. However, an increase in bacterial and viral foodborne disease outbreaks in recent years has been associated with this emerging trend for “healthy eating”. There are good reasons for concern, since many vegetables are eaten raw or only lightly cooked, without a significant pathogen-reducing effect. Moreover, the number of disease cases per outbreak seems to be higher in the case of vegetables than for any other products involved in pathogen transmission^32^.

Soil, manure, and irrigation water could be sources of bacterial pathogens reaching the roots, the leaves, or the fruits. Some bacterial pathogens are found in internal plant tissues, and thus cannot be washed off or killed by disinfectants. *E*. *coli* O157:H7 or *S*. *enterica* have the capacity to colonize the exterior as well as the interior of lettuce and tomato seedlings grown in soil amended with contaminated manure^16, 32–34^. It should be noted that most published studies have been conducted under laboratory growing conditions or inside chambers that could have favored the entry of bacteria into plant tissue. In contrast, an outdoor environment should favor the development of thicker tissues and cuticles that could act as barriers to bacterial penetration. However, indoor cultivation in greenhouses is a common activity in vegetable production and is the only way to produce sprouts, leading to intervention with different treatments to eliminate pathogens^35^.

Even without internalization, the survival of bacterial pathogens on vegetable surfaces and in soil is critical because this is the route of transmission to humans. In the particular case of STEC, additional evaluation of Stx phages —the main drivers behind *stx* transference and the emergence of new virulent STEC strains— is necessary because their capacity to infect and convert new bacterial strains depends on this persistence.

Of particular importance is the occurrence, persistence, and transduction capacity of Stx phages in soil samples. Manure, treated wastewater, and sludge are routinely applied to crop fields as a soil amendment^36^. If the wastes are inadequately treated before land application, then viable pathogens may survive and grow in field soils and contaminate plants^37^. Moreover, the same fields could be used to produce various crops, extending the risk of contaminating different vegetables. While measures and regulations envisage diverse bacterial and viral pathogens, little attention is paid to the presence of phages, compromising the safety of fresh produce.

Our results show that bacteria remained present and cultivable, and phages remained present and infectious in the samples tested after at least, 10 days of storage using two common storage temperatures. The survival of pathogenic STEC bacteria, including O157 serotype, has previously been demonstrated in leafy greens^38–40^ and in manure-amended cropland soil^15, 41, 42^, showing reductions over 10 days in the same range as, or slightly higher than, the ones observed here. The minimal reduction observed at 23 °C could have been balanced by regrowth of bacterial cells during the storage period, but if this is true, then it is assumed that the bacteria could remain metabolically active, hence being even more susceptible to Stx phage conversion. This metabolic activity does not always happen under natural conditions, where the bacteria could be starved or in a viable but not culturable state, rendering phage infection difficult.

However, the aim of this study was not to confirm bacterial stability, which has been demonstrated previously in more extended studies, as indicated above. Rather, the goal was to evaluate how Stx phages behave in these matrices. Our first observation is that Stx phages were present in the different types of vegetable and soil samples. Each sample of our study was collected from a different grocery or field. The diverse origin of the samples allows to get an overview of the occurrence of Stx phages in our area, but did not allow to correlate the presence of Stx phages with a particular site or field since only one sample from each field was analyzed. The ubiquity of free Stx phages, in particular Stx2 phages, in different environments has been extensively reported^43–48^, including in commercial salad samples^45^. In agreement with these studies, they were also detected in the present study, with a higher prevalence of free Stx2 phages than of Stx1 phages^49^, and within an area of study with a relatively low incidence of STEC infections^11^. The second observation is that at least some of the Stx phages in the samples had the capacity to propagate within the matrices, as shown in the experimental approach employed here. Some samples (*e*.*g*. spinachs samples) showed negative when analyzed directly. These negative results could be caused either by the absence of Stx phages or by the presence of low amounts of Stx phages below the detection limit of the method used. We determined that this second possibility was the reason of negative results in many cases, since these negative samples showed detection of Stx phages after they infect and multiply in the *E*. *coli* WG5 strain in the propagation experiments. After propagation, the densities of Stx phages reached in some cases the threshold required for their detection and some of the samples initially negatives appeared positive. The differences between Stx1 and Stx2 phages were again caused by the negative results obtained for Stx1 phages in many samples when directly analyzed. Some samples showed a low increase in Stx phages, that could be attributable to causes other than propagation or to experimental deviations. This could refer to; 1) a fraction of infectious Stx phages that have been randomly adsorbed to particles in the sample and are removed during the steps used for phage purification, 2) Stx phages that being infectious do not propagate efficiently in the host strain used, or 3) Stx phage particles (or generalized transducting particles carrying *stx*)^50, 51^ that are able to attach to and to introduce DNA in the host strain (and therefore are potentially able to generate transductants), but are not able to produce a new phage progeny. It is difficult to establish the minimum fold increase that could determine that propagation has occurred, and very low values of propagation can be doubtful. Nevertheless, the high increase in the GC number observed in many of the samples points towards the capacity of Stx phages to produce new particles after infecting *E*. *coli* WG5 strain, or in lesser extent other bacterial hosts in the matrices. Susceptibility to infection is the first step for *stx* conversion if the right metabolically active host can be reached. This is important because the detection of non-infective, or even defective Stx phages of phage-like particles, unable to transfer the gene as we suspect might sometimes be the case^52^, annuls the risk of *stx* transduction, and hence of the generation of new STEC.

The detection of non-infective Stx phages particles is one explanation of the lower values of infectious Stx phages in comparison with the qPCR values, that does not distinguish between infectious and non-infectious particles. It is also highlighted by the observed lack of correspondence between both results in some of the persistence experiments, in which the conditions could have inactivated the ability of infection of the phages but kept their DNA unaltered, in accordance with previous studies^26^. The fact that some matrices (lettuce or sprouts) do not show correspondence between the reduction of infectious Stx phages and their qPCR values, whereas other matrices show also reduction in qPCR values, opens several possibilities. It can be considered that the characteristics of these particular matrices protect better the capsids and the phage DNA after ten days while affecting the phage attachment and DNA infection required for propagation. On the other hand, it could also be that the characteristics of the matrix could have favoured aggregation of the phage particles, reducing the numbers of PFU and allowing an easier elimination of aggregates through their adsorption on membranes than single virions^53^, but maintaining the GC numbers intact.

As described in previous studies^26, 54^, the persistence of Stx phages to different conditions confirms the role of phages as a reservoir of *stx*. If naturally occurring infectious Stx phages are present in vegetable and soil samples, where they can persist for a certain length of time and maintain the capacity to propagate, the transduction of *stx* to the background flora could, under the right conditions, take place. This should be a rare occurrence, and fortunately the high densities of host bacteria and Stx phages required for *stx* transduction in food matrices shown in previous studies^28^, are not commonly found in food samples. Nevertheless, as demonstrated in our model, there was no restriction on transduction in the matrices analyzed, and new STEC could be generated, in accordance with previous studies on soil^55^, salads^28^, and biofilms^56^. Given that three of our matrices are consumed raw (lettuce, sprouts, and cucumber), and even spinach may be included as a salad ingredient, the low number of transductants generated would be sufficient to pose a health problem.

The outbreak of *E*. *coli* O104:H4 in Europe^22^, in addition to the human casualties and health-associated costs, indicates that accidental STEC contamination or emergence due to Stx phage transduction events could unfortunately become a common occurrence. Protecting vegetables against possible deliberate, accidental, or natural microbial contamination is imperative to ensure a safe food chain. Methods to reduce microbial risks in the vegetable food chain should not neglect Stx phage elimination.

## Methods

### Bacterial strains, bacteriophages, and media

*E*. *coli* C600 (933W∆*stx*_2_::*gfp*::*cat*) (Fig. S1)^57^ and bacteriophage 933W (Fig. S1), induced from lysogenic *E*. *coli* C600 (933W)^58^, were used to evaluate the persistence of STEC and Stx phages in vegetables and soil. *E*. *coli* WG5 (ATCC 700078) was used as the host strain to evaluate the infectivity of Stx phages. *Shigella sonnei* strain 866 (Tc^R^) and recombinant phage 933W (Δ*stx*_2_::*kan*) (this study, Fig. S1) were used to generate lysogens in the different matrices.

Luria-Bertani (LB) broth and agar were used to culture bacteria and when necessary, media were supplemented with chloramphenicol (Cm) (15 µg.ml^−1^), tetracycline (Tc) (10 µg.ml^−1^), and kanamycin (Km) (35 µg.ml^−1^). Phages were enumerated using modified Scholtens Agar (MSA) (ISO 10705-2)^59^ containing 6 mM CaCl_2_ and 0.5% glycerol. Phosphate-buffered saline (PBS) (137 mM NaCl, 8 mM Na_2_HPO_4_, 1.46 mM KH_2_PO_4_, 2.7 mM KCl [pH 7.4]) and phage buffer (PB) (22 mM KH_2_PO_4_, 50 mM Na_2_HPO_4_, 85 mM NaCl, 1 mM MgSO_4_, and 0.1 mM CaCl_2_,) were used to prepare dilutions of bacteria and phages, respectively.

### Samples and procedures

Fifteen lettuce, 15 cucumber, and 15 spinach samples were bought each in a different local supermarket in 2015–2016. A portion of ten g of each vegetal sample was used without further processing for Stx detection, persistence and propagation experiments. For the generation of transductants, to reduce the presence of background flora, samples were rinsed with 200 ml of sterile distilled water containing 2.5 ppm of free chlorine. Excess of liquid was removed from the sample before the experiments. Twenty six soil samples were collected from 6 greenhouses, 5 gardens, and 15 croplands in Barcelona (northeast Spain) (9) and Asturias (north Spain) (6). Ten g of each soil samples were used in all the experiments without further processing. From these, 16 were analyzed fresh for the occurrence of Stx phages and ten were stored at 4 °C for six months to evaluate the persistence of Stx phages after long term storage.

Lentils were sprouted in the laboratory from lentil seeds using a specific germinator (A. Vogel, bioSnacky) (Fig. 4). Seeds were irrigated twice a day with sterile mineral water at 23 °C for 4 days. After this period, sprouts were collected and used as matrices. The procedure was repeated and new sprouts were germinated for each independent experiment. Portions of ten g of sprouts were used without further processing.

For persistence experiments, 3 ml of *E*. *coli* C600 (933W∆*stx*_2_::*gfp*::*cat*) culture at logarithmic phase of growth (*ca* 3 × 10^8^ cells) and 5 ml of phage 933W (containing *ca* 10^8^ pfu of phages) were inoculated in 10 g of each matrix and the samples incubated at 23 °C and 4 °C. The number of bacteria and phages was monitored from the mixture at different times (0, 24 h, 48 h, 72 h, 168 h, and 240 h).

To recover bacteria and phages, each sample was placed together with 20 ml of PBS in stomacher bags with filters (Afora, Barcelona, Spain) and homogenized using a Masticator (IUL Instruments GmbH, Königswinter, Germany) for 2 min. In soil samples, recovery of bacteria was performed as above. For the recovery of Stx phages from soil, in addition to the homogenization with 20 ml of PBS, the pH of the homogenate was increased at 8.5 with NaOH 0.1 N and by adding of 0.5 g of Beef extract (Sigma Aldrich, Ltd) (method adapted from ref. 60).

Bacteria were enumerated by colony counts and molecular detection (*gfp* qPCR)(Table 2). To evaluate Stx phages, the homogenate was filtered using low-protein-binding 0.22 µm-pore-size membrane filters (Millex-GP, Millipore, Bedford, MA, USA). The Stx phages in the filtrate were enumerated in *E*. *coli* WG5 strain and plaque blot and by molecular detection (*stx*_2_ qPCR), as described below.

Transduction experiments in the matrices were performed by inoculating 7 ml of a culture of *S*. *sonnei* strain 866 (Tc^R^) at logarithmic phase of growth (*ca* 5 × 10^8^ cells) and 9 ml of recombinant phage 933W (Δ*stx*_2_::*km*) (Fig. S1) (*ca* 10^8^ phage particles from a purified phage lysate) in 10 g of each matrix at room temperature (23 °C) and incubated for 12 hours. Control was performed with the same volumes of phage and bacteria inoculated in 20 ml of LB broth and incubated under the same conditions. After incubation, the samples were homogenized as described above and the homogenate was used to assess the number of lysogens of *Shigella sonnei* (933W (Δ*stx*_2_::*kan*) generated by plating dilutions of the homogenate in LB agar plates containing Km and Tc. Lysogens were confirmed by PCR using Rho/Km-3 as primers (Table 2). All experiments and conditions were assayed in triplicate or more.

### Preparation of phage lysates

LB cultures of the lysogens *E*. *coli* C600 (933W) or *E*. *coli* C600 (933WΔ*stx*_2_::*kan*) were grown at 37 °C under agitation (180 rpm) to reach an optical density (OD_600_) of 0.3 as measured with a spectrophotometer (Spectronic 501; Milton Roy, Belgium). Freshly prepared mitomycin C (0.5 µg.ml^−1^) was added and the cultures were incubated overnight at 37 °C in the dark in a shaker to induce phages. After incubation, the cultures were filtered through low-protein-binding 0.22-µm-pore-size membrane filters (Millex-GP; Millipore, Bedford, MA, USA) and chloroform treated (1:20) (v:v).

### Construction of the *E*. *coli* C600 (933WΔ*stx*_2_::*kan*) recombinant lysogen

*E*. *coli* C600 lysogenic for phage 933W was used to prepare a recombinant strain in which *kan* (conferring km resistance) cassette replaces a 369 bp fragment of the *stx*_2_ *A* gene (Fig. S1). For this purpose, plasmid pKD46 with the Red recombinase system^61^ was transformed in C600 (933W) strain^58^. A stx_2_A-kan-stx_2_B amplimer was generated by PCR using km5-stx/km3-stx as primers (Table 2) and then purified and electroporated into the laboratory strain *E*. *coli* C600 (933W) containing pkD46 as previously described^62^.

### Phage DNA and total DNA extraction

Phage lysates were treated with DNase (100 units.ml^−1^ of the phage lysate), proteinase K (0.5 μg.ml^−1^), and phenol/chloroform (1:1 *v*/*v*) (Sambrook and Russel, 2001). The mixture was added to Phase Lock Gel Tubes (5- Prime, Hucoa Erlöss, Madrid, Spain) and centrifuged following the manufacturer’s instructions. DNA was precipitated using 100% ethanol and 3 M sodium acetate and resuspended in 50 µl of ultrapure water. Total (bacterial or phage) DNA was extracted from vegetables and soil using a commercial kit, NucleoSpin Blood (Macherey-Nagel, Neander, Germany). DNA was quantified using a Nanodrop ND-1000 spectrophotometer (NanoDrop Technologies, Thermoscientifics, Wilmington, DE).

### Propagation of Stx phages in the matrices

The capacity of Stx2 and Stx1 phages in the samples to infect and propagate using *E*. *coli* WG5 as host was evaluated. Ten samples of each matrix, each sample collected from a different location, were used as replicates of a given type of sample. For each sample, two technical replicates were performed. To this end, one ml of the phage suspensions extracted from the matrix and quantified as described above were used to infect one ml (10^8^ CFU/ml) of a culture containing *E*. *coli* WG5 grown at logarithmic phase in 8 ml of LB and incubated overnight at 37 °C. Phages were extracted from the enrichment and quantified by qPCR after incubation, and the number was compared with the original number of phages in the samples taking into account the dilution factor. Stx phages were considered as infectious when a significant (*P* < 0.05) increase in GC was observed in the supernatant from the enrichment cultures compared to the original homogenate (Fig. 1 positive bars). Stx phages were considered as not infectious when a significant (*P* < 0.05) decrease (Fig. 1 negative bars) or not significant (*P* > 0.05) changes in GC were observed in the supernatant from the enrichment cultures compared to the original homogenate.

In addition, long-term storage was evaluated in ten soil samples. These were stored at 4 °C for six months and Stx phages were extracted from 10 g of each sample as described above and were analyzed directly and after propagation in a *E*. *coli* WG5 culture enrichment.

### Bacterial and phage quantification

Bacteria were monitored by colony counts from decimal dilutions in PBS and inoculation on LB agar supplemented when necessary with Cm, Km, or Tc at different time intervals and temperatures. Incubation was performed at 37 °C for 18 hours.

Infectious phages were evaluated in 1 ml of serial decimal dilutions of the phage suspensions or the homogenates in PBS at different times and temperatures using double-agar layer techniques^59, 63^ and *E*. *coli* WG5 as the host strain. One milliliter of the culture was mixed with MSB soft agar (MSB broth with 0.7% agarose)^59^ supplemented with 6 mM CaCl2 and 0.5% glycerol for optimal visualization of the plaques. After incubation, and since Stx plaques generated by Stx phages are difficult to visualize, plaques were enumerated after plaque blot hybridization performed as described below.

For molecular detection of bacteria and phages, the *gfp* assay (Table 2) was used to detect *E*. *coli* C600 (933W∆*stx*_2_::*gfp*::*cat*), and *stx*_1_ *and stx*_2_ qPCR assays (Table 2) were used to detect Stx phages in the samples and to enumerate phage 933W. Since Stx phages are known to carry only one *stx* copy, each *stx* copy was considered an individual phage particle.

### Plaque blot hybridization

To better quantify the plaques of lysis for detection of the *stx*, plaques were transferred to a nylon membrane (Hybond-N+, Amersham Pharmacia Biotech, Spain) using a standard procedure^64^. Hybridization was performed as previously described (García-Aljaro *et al*.^65^). The nylon membranes were hybridized at 65 °C with a digoxigenin-labeled *stx*_2_ probe^29^. Hybridization was accomplished with the DIG-DNA labeling and detection kit (Roche Diagnostics, Barcelona, Spain), following the manufacturer´s instructions.

### Standard and qPCR procedures

Standard PCRs were performed using a GeneAmp PCR system 2400 (PE Applied Biosystems, Barcelona, Spain). The primers used are described in Table 2. qPCR assays (Table 2) were performed as previously described under standard conditions in a Step One RT PCR System (Applied Biosystems). Genes were amplified in a 20 μl reaction mixture with the PCR Master Mix (Applied Biosystems). The reaction contained 1 or 9 μl of the DNA sample or quantified plasmid DNA. All samples were run in triplicate, as well as the standards and negative controls. GC was defined as the mean of the triplicate data obtained.

### Statistics

Statistical tests were performed using the Statistical Package for Social Science software (SPSS). One or two-way analysis of variance (ANOVA) was used to assess whether the values of each microorganism showed significant reductions at each temperature assayed. A paired Student’s-T test was used to evaluate the differences between Stx phage GC before and after enrichment. For all statistical analyses, P-values below 0.05 were considered significant.

### Data availability statement

All data generated or analysed during this study are included in this published article (and its Supplementary Information files).

## Electronic supplementary material

Figure S1
