# Supplementary material for: Contribution of cropland to the spread of Shiga toxin phages and the emergence of new Shiga toxin-producing strains
Source: Sci Rep. 2017 Aug 10;7:7796. doi: 10.1038/s41598-017-08169-6 (PMC5552810; doi:10.1038/s41598-017-08169-6)
Supplement: Supplementary file 1 — Figure S1 [file 41598_2017_8169_MOESM1_ESM.pdf]

Supplementary material

Contribution of cropland to the spread of Shiga toxin phages and the emergence of new Shiga toxin-producing strains

Pablo Quirós, and Maite Muniesa*

Figure S1.- Genetic organization of the *stx* operon in phage 933W in a Shiga toxin *E. coli* strain serotype C600(933W), in phage 933W after replacing the *stx*$_2$ gene by the km cassette and in phage 933W after replacing the *stx*$_2$ gene by the *gfp-cat* fragment, generating *E. coli* C600 (933Wgfp). The schemes indicate where are located the fragments amplified by the qPCR assays with the respective primers for *stx, km, cat* and *gfp* respectively and their length (bp).

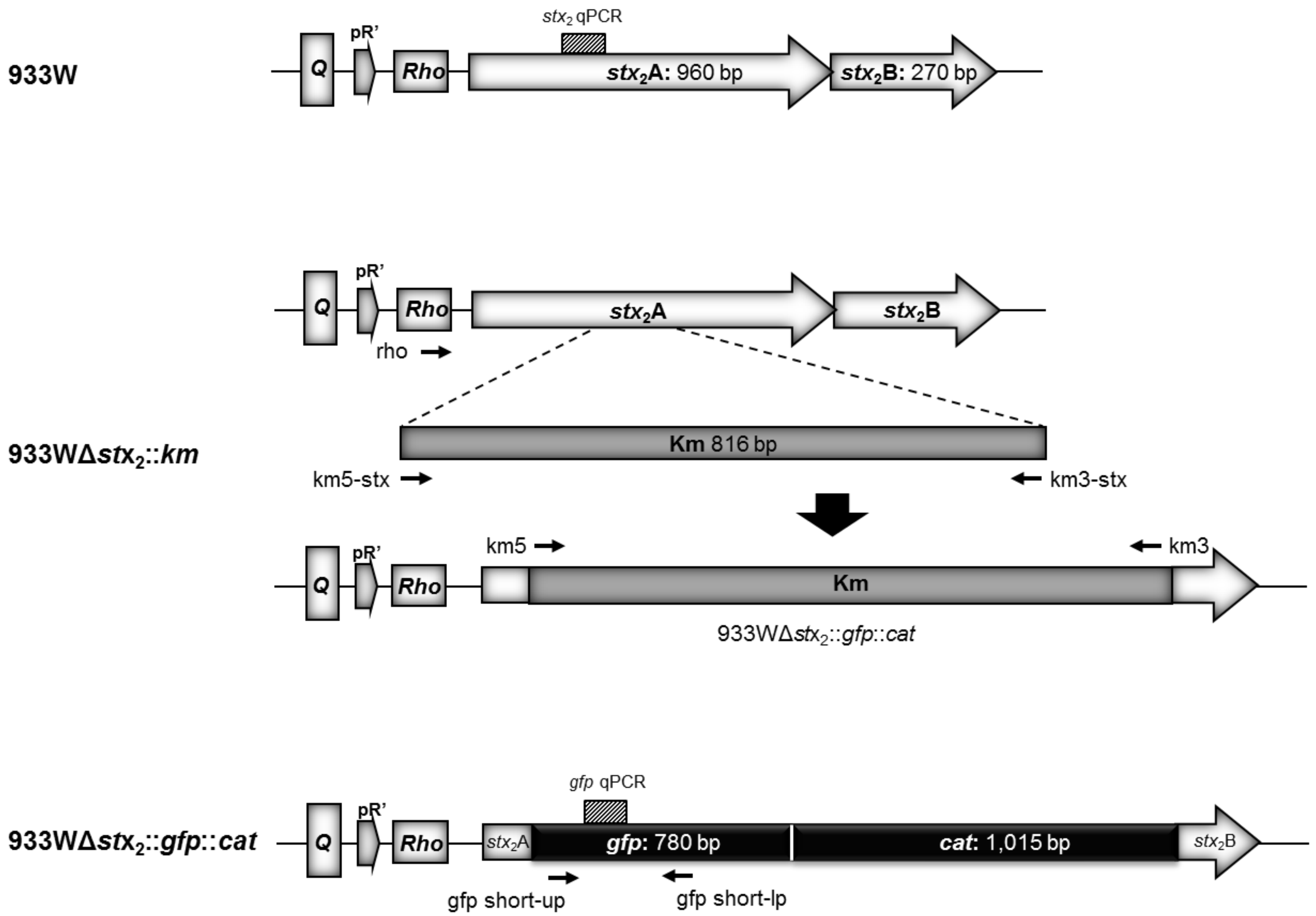
933W
stx_2 qPCR
Q
pR'
Rho
stx_2A: 960 bp
stx_2B: 270 bp
933WΔstx_2::km
stx_2A
stx_2B
rho
Km 816 bp
km5-stx
km3-stx
km5
km3
Km
933WΔstx_2::gfp::cat
933WΔstx_2::gfp::cat
gfp qPCR
stx_2A
gfp: 780 bp
cat: 1,015 bp
stx_2B
gfp short-up
gfp short-lp

Fig. S1
